# ITCZ shift and extratropical teleconnections drive ENSO response to volcanic eruptions

**DOI:** 10.1126/sciadv.aaz5006

**Published:** 2020-06-03

**Authors:** Francesco S. R. Pausata, Davide Zanchettin, Christina Karamperidou, Rodrigo Caballero, David S. Battisti

**Affiliations:** 1 Centre ESCER (Étude et la Simulation du Climat à l’Échelle RÉgionale) and GEOTOP (Research Center on the dynamics of the Earth System), Department of Earth and Atmospheric Sciences, University of Quebec in Montreal, Montreal, QC H3C 3J7, Canada.; 2 Department of Environmental Sciences, Informatics and Statistics, University Ca’Foscari of Venice, Mestre, Italy.; 3 Department of Atmospheric Sciences, University of Hawaii at Mānoa, Honolulu, HI, USA.; 4 Department of Meteorology, Stockholm University and Bolin Centre for Climate Research, Stockholm, Sweden.; 5 Department of Atmospheric Sciences, University of Washington, Seattle, WA, USA.; 6 UNI Research, Bergen, Norway.

## Abstract

The mechanisms through which volcanic eruptions affect the El Niño–Southern Oscillation (ENSO) state are still controversial. Previous studies have invoked direct radiative forcing, an ocean dynamical thermostat (ODT) mechanism, and shifts of the Intertropical Convergence Zone (ITCZ), among others, to explain the ENSO response to tropical eruptions. Here, these mechanisms are tested using ensemble simulations with an Earth system model in which volcanic aerosols from a Tambora-like eruption are confined either in the Northern or the Southern Hemisphere. We show that the primary drivers of the ENSO response are the shifts of the ITCZ together with extratropical circulation changes, which affect the tropics; the ODT mechanism does not operate in our simulations. Our study highlights the importance of initial conditions in the ENSO response to tropical volcanic eruptions and provides explanations for the predominance of posteruption El Niño events and for the occasional posteruption La Niña in observations and reconstructions.

## Teaser

Volcanic eruptions trigger ENSO response through shifts in the ITCZ and extratropical-to-tropical teleconnections.

## INTRODUCTION

The El Niño–Southern Oscillation (ENSO) phenomenon arises spontaneously from the coupling between the atmosphere and the ocean in the tropical Pacific (*1*), and it affects climate worldwide (*2*). Whereas ENSO is considered mainly as an intrinsic mode of interannual-to-decadal tropical climate variability, its evolution can be substantially affected by external forcing factors such as changes in Earth’s orbit or in the atmospheric greenhouse gas composition (e.g., *1*). Here, we focus on the ENSO response to tropical volcanic eruptions and clarify how different response mechanisms are activated depending on the spatial structure of the forcing.
Although there is no consensus yet because of uncertainties in reconstructions of past volcanic forcing (e.g., see Sigl *et al.* (*3*) and references therein), observations (e.g., *3*, *4*) and paleoclimate records (e.g., *5*–*8*) mostly suggest that warm ENSO anomalies, i.e., El Niño events, are likely to develop following major tropical volcanic eruptions. Several modeling studies also show El Niño–like anomalies following a tropical eruption (*5*, *9*–*15*). A few studies display instead cold ENSO anomalies, i.e., La Niña–like events (*16*, *17*), although these may be related to a volcanically induced cooling of the whole tropical belt rather than true (coupled dynamical) La Niña anomalies (*5*, *18*). Dynamical variables point to El Niño–like anomalies developing around 6 to 12 months after the eruption peak (*16*).

Whereas an El Niño–like response is consistently diagnosed in most climate models, the underlying dynamics are unclear and at least four mechanisms have been proposed to explain how tropical volcanic eruptions can project onto the ENSO mode. The first mechanism concerns oceanic processes and is linked to the preferential cooling in the western relative to the eastern Pacific that weakens the zonal sea surface temperature (SST) gradient along the equatorial Pacific. The reduced zonal SST gradient causes a relaxation of the trade winds, which, in turn, yields a temporary weakening of the ocean upwelling in the eastern Pacific that is then amplified by the Bjerknes feedback, leading to an El Niño. This so-called “ocean dynamical thermostat” (ODT) mechanism emerged in simulations with an idealized model and an imposed uniform radiative cooling (*19*, *20*). However, some simulations with full-complexity coupled climate models suggest that the ODT mechanism may be overwhelmed by other feedbacks triggered in the coupled ocean-atmosphere system (*16*). Another suggested mechanism is based on the land-ocean temperature gradient (*10*, *15*), which is enhanced after a volcanic eruption as land [Maritime Continent (*10*)] initially cools faster than the ocean. The increased gradient would then initiate a westerly wind anomaly in the western equatorial Pacific, leading to an El Niño–like anomaly through the Bjerknes feedback (*21*). The third mechanism invokes atmospheric teleconnections associated with an altered Walker circulation caused by the weakening of the West African monsoon following the volcanically induced cooling of tropical Africa as the primary cause of the posteruption El Niño–like anomalies (*5*).

More recently, Pausata *et al.* (*22*) showed that, for large summer high-latitude eruptions in the Northern Hemisphere (NH), the El Niño–like response is caused by the NH cooling that shifts the Intertropical Convergence Zone (ITCZ) southward via energetic constraints (*23*, *24*). The southward shift of the ITCZ weakens the trade winds along the equatorial Pacific, leading to an El Niño–like response through the Bjerknes feedback (*21*). Other studies (*12*, *14*, *25*) consistently suggest that the El Niño–like response following NH tropical eruptions may be initiated by a southward displacement of the ITCZ, while Southern Hemisphere (SH) volcanic eruptions may then favor a La Niña–like response. A related mechanism for the preferential excitation of an El Niño following major volcanic eruptions builds on the recharge-discharge theory of ENSO and includes changes in the wind stress curl during the eruption year as one of the triggering factors (*18*).

The aforementioned studies highlight the importance of the spatial characteristics of the imposed volcanic forcing—especially its cross-equatorial asymmetries over the Pacific region—for the ENSO response. The spatial structure of the volcanic aerosols can contribute to determining the latitudinal position of the maximum surface cooling and, hence, the direction of the meridional shifts of the ITCZ. In turn, meridional shifts in the location of the ITCZ may interfere constructively or destructively with the other abovementioned mechanisms. For example, if the ODT mechanism were to play a role in the ENSO response, in an eruption that resulted in aerosols concentrated in the equatorial region and northward, it would act in synergy with the ITCZ shift to ensure a robust El Niño–like response. On the other hand, the ODT mechanism would damp the ITCZ-driven La Niña–like response in the case the aerosols were mostly confined to the SH. Similarly, the ITCZ shift would constructively superpose on Maritime Continent cooling for tropical NH eruptions, and destructively for tropical SH eruptions. No study has hitherto disentangled the interplay between the ITCZ shift and the other mechanisms determining ENSO evolution in posteruption years. Sensitivity experiments designed upon the constructive/destructive superposition of competing mechanisms dependent on the spatial structure of the forcing could be illuminating regarding the relative strength of such mechanisms.

**Fig. 1:**
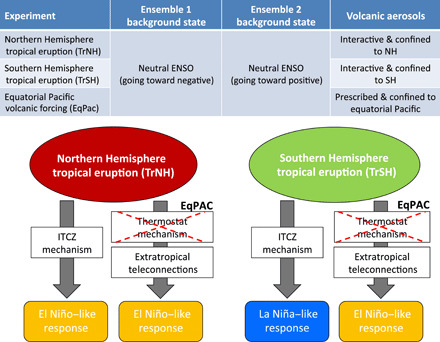
Schematic description of the experiments and the mechanisms affecting ENSO in our simulations. Schematic description of the experiments and representation of the potential impacts on ENSO of the ITCZ shift, ODT mechanisms, and extratropical teleconnections following volcanic eruptions.

**Fig. 2:**
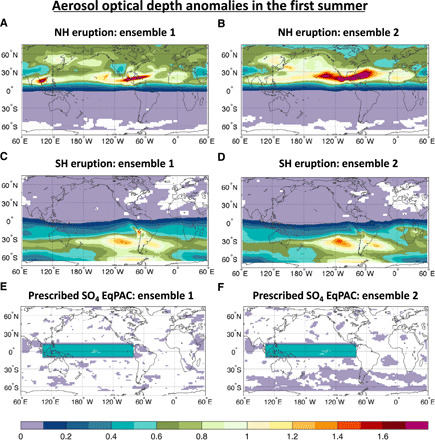
Changes in aerosol optical depth. Anomalies in aerosol optical depth (at 550 nm) for the summer (June to September) following the eruption for the TrNH (**A** and **B**), TrSH (**C** and **D**), and EqPAC (**E** and **F**) experiments relative to the no-volcano simulations.

**Fig. 3:**
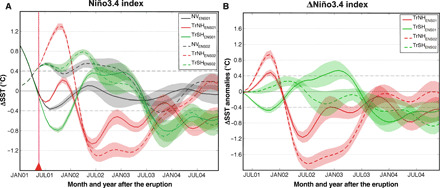
Niño3.4 index and its changes. (**A**) Niño3.4 index for the TrNH and TrSH volcano and no-volcano simulations. In (A), the triangle and the vertical red line highlight the onset of the eruption. (**B**) Niño3.4 index anomalies (volcano minus no-volcano experiments) for TrNH and TrSH cases. In (B), the anomalies are shown starting from the month of the eruption. The lines represent the ensemble mean, and the shadings represent the SE of the ensemble mean.

Here, we design and perform a series of sensitivity experiments to quantify the ENSO response to tropical volcanic eruptions and, in particular, to illuminate the processes that govern the response as a function of the hemispheric asymmetry in the aerosol forcing. Volcanic eruptions resulting in symmetric aerosol distributions rarely occur due to the strong hemispheric asymmetry in the stratospheric (Brewer-Dobson) circulation (*26*) and preclude a clear quantification of the ITCZ shift mechanism in driving the ENSO response. Therefore, we perform ensemble simulations of boreal summer eruptions occurring in the tropical Northern (TrNH) and Southern Hemisphere (TrSH) (Fig. 1; see Materials and Methods) using an Earth system model with an aerosol module embedded [Norwegian Earth System Model: NorESM1-M (*27*, *28*)]. The choice of the volcanic eruption locations ensures that the volcanic aerosol loading is primarily confined to one hemisphere. However, both TrNH and TrSH eruptions result in a large amount of aerosol over the equator, which could potentially trigger the ODT mechanism. The ENSO response to heterogeneous radiative forcing of such hemispherically asymmetric aerosol distribution (Fig. 2 and fig. S1) allows us to evaluate the importance of the ITCZ shift caused by the differential hemispheric cooling relative to other mechanisms such as the ODT mechanism. We select two different initial conditions for the ENSO state (see Fig. 1 and Materials and Methods), each yielding a 20-member ensemble (namely, ENS01 and ENS02) for each “volcano” case (TrNH and TrSH) as well as the unperturbed “no-volcano” (NV) case. The unperturbed cases share identical initial conditions as the volcano members, but with no volcanic eruption. The initial states were chosen as representative of evolution toward a weak warm and weak cold ENSO phase in the absence of the eruption (Fig. 3 and fig. S2) to account for the uncertainty due to dependency of ENSO response on the background climate state at the time of the eruption (*10*, *15*, *29*).

## RESULTS

### ITCZ shift and ODT mechanisms

**Fig. 4:**
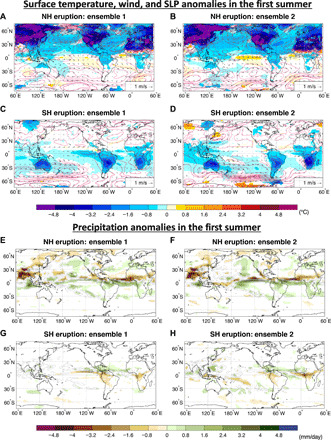
Changes in surface temperature, wind, sea level pressure (SLP), and precipitation. Changes in surface temperature (°C, shadings), wind (m/s, arrows), SLP (hPa, contours) (**A** to **D**) and precipitation (mm/day) (**E** to **H**) in the first summer (June to September) following the TrNH (A, B, E, and F) and TrSH (C, D, G, and H) eruptions for each ensemble. Only temperature and precipitation values that are significantly different at the 5% level using a local (grid-point) *t* test are shaded. The contours follow the color bar intervals (solid for positive and dashed for negative anomalies; the zero line is omitted).

The ENSO response following tropical volcanic eruptions clearly differs depending on whether the aerosol remains restricted to the NH or the SH. For TrNH, the equatorial central Pacific SST immediately responds with clear El Niño–like anomalies, while for TrSH, the response is ambiguous: The eruption triggers either La Niña–like anomalies or weak El Niño–like anomalies in the first fall/winter following the eruption, depending on the initial conditions at the time of the eruption (Figs. 3 and 4 and figs. S3 and S4).

The SST anomalies in the summer of the eruption show that for TrNH, there is a stronger cooling in the western compared with the eastern equatorial Pacific (Fig. 4, A and B), while zonal differences are subtler or absent in TrSH (Fig. 4, C and D). The precipitation anomalies clearly indicate a southward shift of the ITCZ for TrNH (Fig. 4, E and F, and table S1), and a weaker northward ITCZ shift for TrSH (Fig. 4, G and H, and table S1), pointing to the ITCZ shift as a key player in the ENSO response (see also fig. S4). In both experiments, the zonally averaged ITCZ is shifted away from the hemisphere that is cooled (i.e., where the volcanic aerosol is confined) as expected based on energetic constraints (*23*, *24*).

According to the ODT mechanism, a uniform negative radiative forcing over the equatorial Pacific would cause a preferential cooling of the western Pacific warm pool, reducing the east-to-west SST gradient in the equatorial Pacific and decreasing the trade winds (*20*). In addition to the ODT, the teleconnections associated with the volcanically induced cooling of northern tropical Africa (*5*) and of the Maritime Continent (*10*, *15*) would also favor an El Niño–like response following tropical eruptions; hence, ocean dynamics (the ODT) and atmospheric teleconnections would play in synergy with the ITCZ shift to amplify the El Niño–like response in the TrNH simulations. In the TrSH experiments, while the volcanically induced land cooling is negligible, the ODT mechanism can still be activated and, hence, damps the La Niña–like response (Fig. 1). This appears consistent with the changes in the Niño3.4 index shown in Fig. 3 and the ITCZ shifts that can be seen in Fig. 4 (E to H) and table S1.

As no volcanic aerosol is present in the NH following TrSH eruptions, the land cooling of the Sahel in northern Africa (Fig. 4, C and D) is not only modest but also dynamically induced by the northward shift of the ITCZ, rather than by direct radiative forcing as for the TrNH eruptions. On the other hand, cooling of the Maritime Continent is very similar in both TrSH ensembles (cf. Fig. 4, C and D), while the ENSO response is different (Fig. 3), suggesting that this mechanism is a secondary player in affecting posteruption ENSO evolution. Hence, the ODT mechanism seems to be the only one that can counteract the La Niña–like response induced by the ITCZ displacement in TrSH eruptions.

**Fig. 5:**
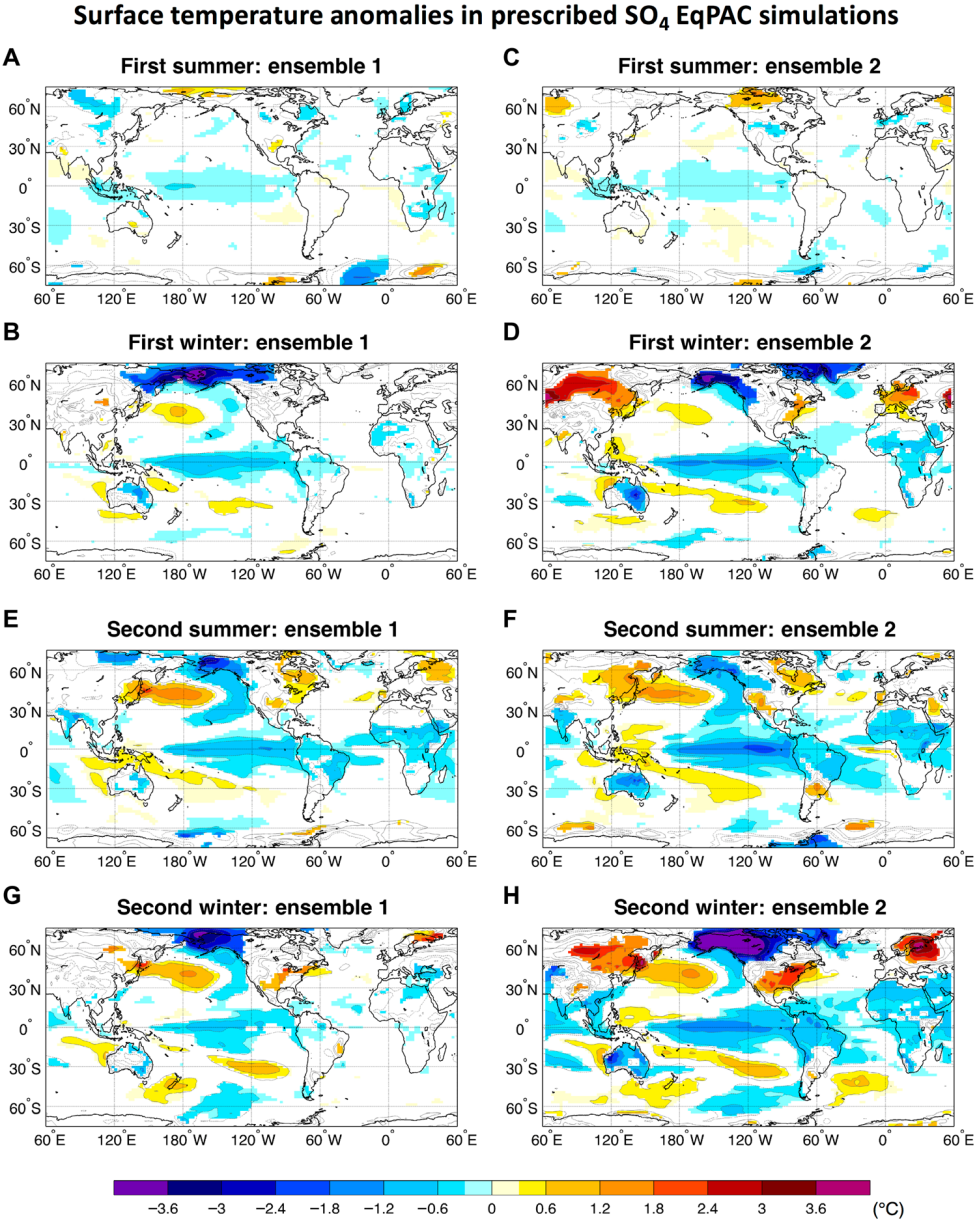
Changes in surface temperature in EqPAC experiments. Surface temperature (°C) changes in the first and second summer (June to September (**A**, **C, E**, and **F**) and winter (December to February) (**B**, **D, G**, and **H**) following the EqPAC volcanic forcing for each ensemble. Only values that are significantly different at the 5% level using a local (grid-point) *t* test are shaded. The contours follow the color bar intervals (solid for positive and dashed for negative anomalies; the zero line is omitted).

To verify this hypothesis and, hence, isolate the ODT from the ITCZ shift mechanism, we follow the original design of the ODT experiment (*20*) and simulate a highly idealized case in which the volcanic aerosols are confined to a box enclosing only the equatorial Pacific (EqPAC; 10°S to 10°N; 100°E to 80°W). In doing so, we do not simulate a volcanic eruption but generate instead a uniform negative radiative forcing over the core ENSO region (Fig. 2, E and F). As the forcing is restricted to the equatorial Pacific, only the ODT mechanism can be triggered with no or only negligible influences from other mechanisms. To increase the signal-to-noise ratio in the response, we set the amplitude of the aerosol forcing over the equatorial Pacific in the EqPAC ensemble to be twice as large as that which resulted from the TrNH and TrSH experiments for the same region (see fig. S1G). The EqPAC simulations yield a weak cooling that is nearly pan-Pacific and projects onto the ENSO mode to trigger La Niña–like anomalies in the winter following the eruption (Fig. 5). This is the opposite of the El Niño–like response expected from the ODT mechanism. In the eastern equatorial Pacific, the strong cooling detected around the thermocline (fig. S3, E and F) also contrasts with the expected subsurface warming from an active ODT mechanism. Both McGregor and Timmermann (*16*) and Stevenson *et al.* (*18*) pointed out that an initial eastern Pacific cooling acts to generate a delayed ocean dynamical response through the subtropical wind stress curl, so that El Niño–like anomalies develop only in the second winter following the eruption. In our EqPAC experiments, however, the second winter remains characterized by La Niña–like anomalies, thus not supporting the delayed ocean dynamical response (Fig. 5).

### Extratropical responses

**Fig. 6:**
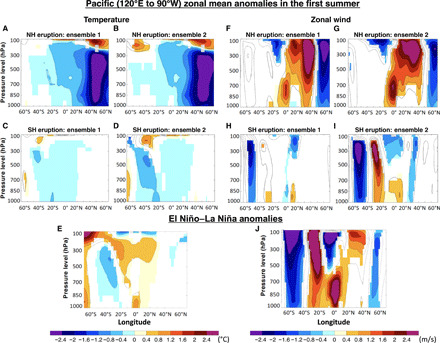
Pacific zonal mean temperature and zonal wind anomalies. Zonal mean atmospheric temperature (°C) (**A** to **D**) and zonal wind (m/s) (**F** to **I**) anomalies over the equatorial Pacific region (120°E to 90°W) in the summer (June to September) following the TrNH (A, B, F, and G) and TrSH (C, D, H, and I) eruptions for each ensemble. Bottom panels show the difference between El Niño and La Niña composite for temperature (**E**) and zonal wind (**J**). Only values that are significantly different at the 5% level using a local (grid-point) *t* test are shaded. The contours follow the color bar intervals (solid for positive and dashed for negative anomalies; the zero line is omitted).

The ENSO response to both TrNH eruptions and to the TrSH_ENS01_ eruption is consistent with the ITCZ shift mechanism: An El Niño–like response develops in the TrNH simulations because of an equatorward shift of the ITCZ, and a La Niña–like response develops in the TrSH_ENS01_ experiment because of a northward shift of the ITCZ. However, the TrSH_ENS02_ simulation shows a weak El Niño–like response, in contrast with an expected La Niña–like response if prompted by the ITCZ shift alone. Because we have shown that the ODT mechanism and the other abovementioned mechanisms (Maritime Continent and tropical Africa cooling) cannot explain the posteruption ENSO evolution (in particular for TrSH experiments), other previously unidentified mechanisms must be at play. In particular, we noted that the volcanically induced radiative forcing can produce an extratropical circulation response that may, in turn, affect the tropics. Extratropical responses to radiative forcing—including meridional shifts in jets and storm tracks—are complex and often consist of a “tug of war” between opposing effects (*30*). In both TrNH and TrSH, the zonal mean temperature response (Fig. 6, A to D) shows a warming of the lower stratosphere at high latitudes (as expected from increased solar absorption by the volcanic aerosol) and a cooling of the underlying troposphere, linked to reduced solar absorption at the surface. The former effect is expected to shift the jet equatorward, while the latter would shift it poleward (*31*). In addition, volcanic forcing leads to a marked cooling of the continents relative to the ocean in all our simulations (Fig. 4, A to D); this can be expected to lead to an anticyclonic response over the continents and a cyclonic response over the oceans, with consequent weakening of the subtropical high and an equatorward shift of the jet in the Pacific (*32*).

As it turns out, the dominant response in our simulations is a poleward shift of the Pacific jet (Fig. 6, F to I) and a strong cyclonic surface pressure anomaly over the midlatitude to subtropical Pacific basin (Fig. 4, A to D). This response is seen in both TrNH and TrSH simulations, although with considerably higher amplitude in the TrNH case. This difference in the magnitude of the response between the TrNH and the TrSH simulations can be attributed to (i) the different magnitude of the radiative forcing due to the season of the eruption (summer in the NH and winter in the SH) (fig. S1) and (ii) the much larger area covered by landmass (reduced heat capacity) in the NH compared with the SH (greater cooling in the NH than in the SH; see Fig. 4, A to D).

One question to address is whether the changes in meridional temperature gradient and jet detected in the simulations are a consequence of the ENSO response to the eruption, rather than a contributor to such response. In the TrNH simulations, the strengthening of the NH jet stream resembles the changes induced by El Niño events in the NH, albeit with a much larger magnitude (cf. Fig. 6, F and G to J). In the TrSH_ENS01_ experiment, if the response of the jet stream was a result of—and not a contributor to—the tropical response (cooling of the equatorial Pacific), then one would expect to see changes in the jet similar to those expected for a La Niña event. However, the jet changes in TrSH_ENS01_ are opposite to those associated with a La Niña event (cf. Fig. 6, H and I to J). In the TrSH_ENS02_ simulation, the jet changes are also El Niño like, but much stronger than in the TrSH_ENS01_ experiment (cf. Fig. 6, H and I). This indicates that the extratropical changes are a direct response to the volcanic forcing rather than driven by ENSO, as evidenced by the lack of significant anomalies in SST and precipitation in boreal summer (from June to September - JJAS) in the TrSH_ENS02_ experiment (Figs. 3B and 4, D and H). To further corroborate that the extratropical changes are a direct response to the volcanic forcing and not mediated by oceanic changes, we performed an additional set of experiments in which we simulate the same volcanic eruptions (TrNH and TrSH) but prescribe the SST to evolve identical to that in the unperturbed coupled simulations. In the prescribed SST experiments, the changes in both the zonal mean temperature and zonal wind due to the volcanic eruptions resemble the anomalies simulated in the fully coupled experiments (cf. Fig. 6 and fig. S5). The changes in the surface fields [sea level pressure (SLP) and wind] forced by the eruptions are also very similar in the fully coupled and prescribed SST simulations (cf. Fig. 4 and fig. S6). Hence, these experiments clearly support our hypothesis that the extratropical responses in the summer of the eruption are almost entirely because of the direct volcanic forcing.

The atmospheric circulation response to the TrNH eruptions features anomalies in the North Pacific that are reminiscent of the circulation anomalies associated with the positive phase of the internally generated Pacific Meridional Mode (PMM) and are similar to the SLP and surface wind anomalies of the summer preceding an El Niño event (cf. Fig. 4, A and B, and fig. S8). A positive phase of the PMM has been shown to be associated with El Niño conditions 6 months later in boreal winter (*33*). The PMM is an effective conduit for extratropical atmospheric influence on ENSO (*34*). However, the cyclonic SLP anomaly in the central north Pacific is shifted slightly northward of the circulation anomalies associated with the PMM, and so, it is not clear how much the volcanically induced extratropical circulation anomalies contribute to the ensuing El Nino-like anomalies in the TrNH eruptions.

In the TrSH eruptions, the change in the strength of the jet at 200 hPa around 30°S to 40°S (Fig. 6, H and I) is associated with a decrease in SLP in the subtropical region around 25°S to 40°S (Fig. 4, C and D). The SLP anomalies extend to the equatorial region in TrSH_ENS02_ (Fig. 4D) and are responsible for the westerly wind anomalies along the central western Pacific (Fig. 4D). The associated slowdown of the trades effectively counteracts their strengthening due to the northward displacement of the ITCZ. In TrSH_ENS01_, instead, the westerly wind anomalies do not properly develop (Fig. 4C) because of the weaker SLP anomalies relative to TrSH_ENS02_ (Fig. 4D), and a La Niña–like anomaly can take place.

That is, in the TrNH simulations, both the extratropical circulation anomalies and the southward ITCZ shift act in the same direction, both favoring development of El Niño conditions. Hence, NH aerosol loading should always generate El Niño–like anomalies due to the constructive interference of the ITCZ shift and the extratropical teleconnections (Fig. 1, bottom left). In contrast, in the case of SH volcanic loadings, there is destructive interference between the energetic constraints requiring the zonally averaged ITCZ to move northward (leading to a La Niña–like response) and the teleconnections from the extratropics favoring the development of El Niño (Fig. 1, bottom right). The end result from these SH volcanic loadings is a tug of war between the ITCZ and the extratropical forcing modulated by the initial conditions of the tropical Pacific (and thus the strength of the already established extratropical conditions before the eruption happens), as will be discussed in the following section.

### The impacts of different initial conditions

As discussed above, the intensity in the teleconnections from the extratropics between ENS01 and ENS02 is different. In both ensembles, one would expect a direct volcanically forced response in the extratropical circulation that would give rise to El Niño–like anomalies and an indirect response that would tend to move the zonal average ITCZ away from the hemisphere that is cooled. However, the different initial conditions may make the climate system more prone to develop El Niño conditions or may lead to a slightly different aerosol forcing distribution and, hence, different extratropical volcanic forcing (Figs. 2 and 6).

While the Niño3.4 index is close to neutral at the time of the eruption for both ensembles, it is evident that the tendency of SST anomalies in the equatorial Pacific—before the eruption occurs—is opposite for the two ensembles (Fig. 3). More specifically, NV_ENS01_ is already evolving toward negative Niño3.4 anomalies and may be less prone to develop westerly wind anomalies along the equator: If so, the ITCZ shift would prevail. On the other hand, NV_ENS02_ is evolving toward positive Niño3.4 anomalies and may be more prone to develop westerly anomalies potentially counterbalancing the effect of the ITCZ shift.

The different initial conditions lead to a different aerosol distribution with higher concentration over the Pacific Ocean in ENS02 than in ENS01 (Fig. 2). While the volcanically induced changes in zonally averaged temperature are very similar in ENS01 and ENS02, notable differences exist over the Pacific (cf. Fig. 6 and fig. S9). These different temperature changes aloft may also contribute to shaping the atmospheric circulation changes in the extratropics to resemble the typical situation of a developing El Niño event more in ENS02 than in ENS01 (cf. Figs. 4, A to D, and 6). This supports our hypothesis that the weak El Niño–like response in the TrSH_ENS02_ is partially due to the fact that the northward shift of the ITCZ is not able to overcome the extratropical forcing that favors the development of an El Niño event in TrSH_ENS02_ while it is in the TrSH_ENS01_ experiment. In a similar fashion, the El Niño–like anomalies are stronger in TrNH_ENS02_ than in TrNH_ENS01_ because of a potentially stronger extratropical forcing in the TrNH_ENS02_.

Furthermore, the initial response of the equatorial Pacific may feed back into the midlatitudes. Hence, teleconnections from the tropics into the extratropics also need to be accounted for, which likely depend on the initial ENSO state and/or the spatial aerosol distribution. Such tropical-to-extratropical teleconnections can then modify the direct extratropical forced response. In the TrNH_ENS01_, the El Niño–like anomalies are weaker in JJAS compared with TrNH_ENS02_ and, thus, so are the potential teleconnections from the tropics into the extratropics. As a consequence, in TrNH_ENS02_, the directly forced extratropical teleconnections will be enhanced by the tropical-to-extratropical teleconnections more than in the TrNH_ENS01_. This may explain then the greater El Niño–like response in the TrNH_ENS02_ during winter relative to TrNH_ENS01_ (Fig. 2). In a similar way, La Niña–like anomalies are already developing in JJAS in TrSH_ENS01_, whereas there are no significant SST anomalies in the equatorial Pacific in TrSH_ENS02_ during that period. Consequently, tropical-to-extratropical teleconnections (La Niña like) weaken the volcanically induced direct extratropical response (El Niño like) in TrSH_ENS01_, while only the volcanically induced direct extratropical response (El Niño like) is expected in the TrSH_ENS02_ simulation. Hence, in the TrSH_ENS01_ experiment, the northward ITCZ shift wins out on the extratropical forcing leading to La Niña–like anomalies, while the extratropical forcing is larger in the TrSH_ENS02_ than in the TrSH_ENS01_ experiment and cancels the effects of the ITCZ displacement.

Further modeling studies are needed to test this mechanism as potential biases in the volcanically induced changes in the atmospheric circulation in the troposphere may have been introduced by a poor representation of the circulation in the stratosphere (see Materials and Methods).

## DISCUSSION

In this study, we designed and performed a set of experiments with an Earth system model to illuminate the short-term response of ENSO to volcanic forcing. Our results demonstrate that the ENSO response is due to the combined impact of two processes: (i) a meridionally displaced ITCZ due to hemispherically asymmetric cooling and (ii) extratropical responses affecting the tropics. In response to NH eruptions, the ITCZ is displaced meridionally toward the equator and, hence, leads to El Niño–like response anomalies 4 to 6 months later. On the other hand, SH eruptions shift the ITCZ away from the equator and, hence, trigger a La Niña–like response. Eruptions in either hemisphere change the meridional temperature gradient and the land-ocean temperature contrast in the extratropics of that hemisphere, causing a regional atmospheric response in the extratropical Pacific that favors the development of El Niño conditions. Hence, summertime NH eruptions robustly cause El Niño–like anomalies about 6 months later, while the response to a summertime SH eruption depends on which of the two processes is dominant. In both hemispheres, the amplitude of the ENSO response depends on the initial conditions in the tropical Pacific and the spatial structure of the applied volcanic forcing, which is also affected by the initial conditions.

These results demonstrate the importance of the ITCZ shift in triggering an ENSO response not only in high-latitude eruptions (*22*) but also in tropical eruptions in which an asymmetric cooling of NH and SH takes place, supporting previous hypotheses (*12*, *14*, *25*). Our results point to a critical dependence of the response on the spatial structure of the forcing, especially interhemispheric asymmetries.

Our results also identify a new mechanism linked to teleconnections from the extratropics, whereby the extratropical response to volcanic aerosol radiative forcing mediates the ENSO response (Fig. 6). Specifically, the volcanically induced changes in meridional temperature gradient and in land-ocean temperature contrast affect the jet stream strength and position and weaken the oceanic subtropical high-pressure systems over the Pacific Ocean (fig. S5, A to D). The weakened subtropical high then causes westerly wind anomalies along the equatorial Pacific that instigate the ENSO anomalies (fig. S5, E to H). Hence, in our simulations, the ITCZ and the teleconnections from the extratropics dominate the posteruption ENSO evolution. While the teleconnection from volcanically induced cooling of tropical Africa can still affect ENSO in the TrNH simulations, the Maritime Continent seems to have a marginal role. The so-called ODT mechanism (*20*) instead is not active in our simulations. This was clearly evidenced in the sensitivity experiment where a homogeneous volcanic forcing is applied over the equatorial Pacific. This experiment develops surface and subsurface ocean anomalies corresponding to a La Niña–like response, rather than to an El Niño–like response as expected from the ODT mechanism. The ODT mechanism may still hold for more idealized setups [e.g., (*16*)] or other types of forcing (e.g., greenhouse gasses). However, further studies with other climate models testing the ODT mechanism and the teleconnections from the extratropics are necessary to evaluate the robustness of our results.

Last, our results provide an explanation for both the predominance of posteruption El Niño events and the occasional posteruption La Niña and neutral events in observations and paleoclimate reconstructions. However, the ENSO responses discussed in this study should only be interpreted as anomalies (El Niño–like or La Niña–like anomalies; Fig. 3B), i.e., intrinsic variability evolving toward a La Niña at the time of the eruption would not necessarily lead to a posteruption El Niño event even for a Northern Hemispheric tropical eruption, but rather a dampening of the ongoing La Niña (see TrNH_ENS01_ in Fig. 3A). Our results highlight that intrinsic variability determined by the selected initial conditions can significantly influence the anomalies induced by the applied forcing. This confirms recent suggestions about the need to better constrain the initial state for an accurate simulation and attribution of posteruption climate anomalies (*35*).

## MATERIALS AND METHODS

### Model description

We used the Norwegian Earth System Model [NorESM1-M (*27*, *28*)], which has a horizontal resolution of 1.9° (latitude) × 2.5° (longitude) and 26 vertical levels. NorESM1-M uses a modified version of Community Atmospheric Model version 4 [CAM4 (*36*)], CAM4-Oslo, to simulate the atmospheric circulation with an updated module that simulates the life cycle of aerosol particles, and primary and secondary organics. NorESM1-M includes treatment of the direct effect of aerosols and the first and second indirect effects of aerosols on warm clouds (*37*). The atmospheric model is coupled to the Miami Isopycnic Coordinate Ocean Model (MICOM), which has a horizontal resolution of ~1.125° along the equator and 53 vertical levels. A detailed description of the model used in this study can be found in Bentsen *et al.* (*27*) and Iversen *et al.* (*28*).

### Experimental design

In the experiments used in the present study, a tropical eruption has been simulated in which 60 Tg of SO_2_ was injected mostly between ~15 and ~21 km (upper troposphere/lower stratosphere) over a period of 3 days to mimic a Tambora-like eruption. Although the injection height was likely much higher than what we used [likely more than 40 km (*38*)], we lowered it to overcome an increased residence time of the aerosol particles (for details, see the Supplementary Materials).

We then performed two experiments starting from a particular instant in time in the control run (1911–1964): 1 June 1923. In one experiment, a volcanic eruption was simulated in the subtropics (17°N) of the NH (TrNH), and in the other experiment, an eruption was simulated in the subtropics (17°S) of the SH (TrSH; see Fig. 1). Each experiment included an ensemble of 20 simulations, each with a small stochastic perturbation applied to the otherwise identical initial conditions. The perturbation, of the order of 10^−14^°C, was applied to the surface temperature. A third experiment was an ensemble of 20 simulations—also constructed starting from 1 June 1923 and with identical initial conditions as the TrNH and TrSH members—but with no volcanic eruption. We distinguished the impact of the volcano from intrinsic noise by comparing the 20-member average of each volcano experiment to the 20-member average of the control. Each ensemble member was run for 4 years, ending on 31 May 1927.

We chose 1 June 1923 as our starting date because the tropical Pacific is in an ENSO neutral state (Niño3.4 index = −0.1°C in June), but in the absence of an eruption is trending to La Niña conditions 3 months later (Niño3.4 index = −0.4°C in September; Fig. 3). To explore the sensitivity of the response to the volcanic eruption to the particular phase of ENSO, we constructed two additional experiments with identical NH and SH tropical eruption emission scenarios, but starting from a different year in the control simulation together with the reference NV experiment (1 June 1927). Unlike for 1 June 1923, the June 1927 volcanic eruption occurs when the ENSO is in a slightly warm state (Niño3.4 index = +0.4°C), and in the absence of a volcanic eruption, it would have remained warm for the next 18 months (see Fig. 2). We denote the three experiments (two volcano and one NV) that start on 1 June 1923 (1 June 1927) as the ENS01 (ENS02) set of experiments. Early summer (1 June) eruptions have been chosen since it is the ENSO developing season, and it has been shown that the associated radiative forcing is most likely to affect the ENSO response (*15*).

Every volcanic eruption shares an identical sulfate emission scenario, which is tailored after the Tambora eruption of 1815—the largest tropical eruption in the past 500 years (*39*).

This experimental design with interactive aerosols allows investigating the two-way interaction between aerosol and climate to explore the associated feedbacks in a more realistic framework compared with approaches where there is a one-way effect of aerosols on climate: While focusing on the climate response, we consider the whole process of forcing generation. This setup, thus, allows to demonstrate that volcanic forcing asymmetries around the equator affect the spatial structure of the forcing as well as the magnitude of the forcing itself, yielding a more realistic quantification of associated climatic impacts.

We have also performed a highly idealized experiment to test the ODT, in which the SO_4_ mixing ratio has been prescribed exclusively over the equatorial Pacific (10°S to 10°N; 100°E to 80°W). The prescribed SO_4_ mixing ratio for the EqPAC simulations has been created to have a peak anomaly in radiative forcing in July over the equatorial Pacific similar to the asymmetric simulations but with approximately double their strength (fig. S1). The prescribed SO_4_ gradient at the borders of the forcing region (equatorial Pacific) is not dissimilar from the gradients that develop in experiments where the volcanic plume is allowed to evolve [see Fig. 2, A and B, in the TrNH eruptions]. Therefore, it does not, in itself, present an additional or unique unrealistic forcing that could unduly affect the results.

To test whether the ODT mechanism is at play in response to a tropical volcanic eruption, we prescribed an aerosol forcing that is symmetric about the equator in the tropical Pacific rather than simulate a volcanic eruption at the equator. This experimental design precludes extratropical forcing and hemispheric asymmetry in forcing that would accompany any tropical eruption by way of the Brewer-Dobson circulation (*26*) and, thus, isolates the impact of the ODT.

The volcanically induced anomalies (Δvolc) are calculated as the difference between the climate state induced by the eruption (V_ENS01_ or V_ENS02_) and the unperturbed climate state (NV_ENS01_ or NV_ENS02_): Δvolc* = V_ENS0_* – NV_ENS0_*, where * stands for the ensemble identifier (1 or 2). Each mean climate state (V_ENS0_*, NV_ENS0_*) is defined for each given ensemble (i.e., the averages of TrNH_ENS01_, TrNH_ENS02_, TrSH_ENS01_, TrSH_ENS02_, EqPAC_ENS01_, EqPAC_ENS02_, NV_ENS01_, or NV_ENS02_) as the ensemble average of all its members.

We have tested the ability of NorESM in simulating the SO_4_ peak concentration and e-folding time against the Pinatubo eruption (June 1991), which is currently the best observed large tropical eruption (see the Supplementary Materials and fig. S10). NorESM is able to reproduce the main features of the Pinatubo eruption, which is sufficient for the scope of this study, that is, to delve into the mechanisms that trigger an ENSO response following a uniform radiative forcing.

Although each volcanic eruption shares an identical sulfate emission scenario, the amount of SO_4_ produced and the strength of the forcing are larger for the TrNH eruptions (Fig. 1 and fig. S1). This is because the eruption occurs during the boreal summer, when higher concentrations of OH radical are available to turn SO_2_ gas into sulfate aerosol, as compared with winter.

The general validity of the conclusions presented in this study must account for possible limitations of our experimental design, in particular (i) the representativeness of the chosen forcing as the 1815 Tambora eruption corresponds to a strong and rare volcanic event and (ii) the limitations of the single model used here and its skill in simulating ENSO and the mean climate state (i.e., climatological biases). The 1815 Tambora eruption is a test case to study the climatic response to volcanic forcing (*35*, *40*, *41*). While we expect the separation between different mechanisms highlighted in this study to be affected by a lower signal-to-noise ratio, they should still be applicable for more frequent but weaker eruptions, such as the 1991 Pinatubo.

NorESM is among the best coupled climate models to represent ENSO concerning the mean climate state of the tropical Pacific and the spectrum of ENSO variability (*42*). It is also weakly affected by the double ITCZ issue as the double ITCZ is less pronounced in NorESM than in other climate models (*27*, *43*).

### Methodological approach

#### ENSO analysis

The ENSO index used in this study is based on monthly SST anomalies averaged over the Niño3.4 region (5°N to 5°S; 170°W to 120°W). We apply a 5-month running mean to remove intraseasonal variations in SST. An El Niño event is defined when the Niño3.4 index exceeds 1 SD (+0.4°C) for at least 6 consecutive months. Unless otherwise noted, all differences discussed in this study are significant at the 95% confidence level using a Student’s *t* test.

## Supplementary Material

aaz5006_SM.pdf

## SUPPLEMENTARY MATERIALS

Supplementary material for this article is available at http://advances.sciencemag.org/cgi/content/full/6/23/eaaz5006/DC1
