# Supplementary material for: ITCZ shift and extratropical teleconnections drive ENSO response to volcanic eruptions
Source: Sci Adv. 2020 Jun 3;6(23):eaaz5006. doi: 10.1126/sciadv.aaz5006 (PMC7269674; doi:10.1126/sciadv.aaz5006)
Supplement: aaz5006_SM.pdf [file aaz5006_SM.pdf]

Science Advances
AAAS

advances.sciencemag.org/cgi/content/full/6/23/eaaz5006/DC1

Supplementary Materials for

ITCZ shift and extratropical teleconnections drive ENSO response to volcanic eruptions

Francesco S. R. Pausata*, Davide Zanchettin, Christina Karamperidou, Rodrigo Caballero, David S. Battisti

*Corresponding author. Email: pausata.francesco@uqam.ca

Published 3 June 2020, *Sci. Adv.* **6**, eaaz5006 (2020)
DOI: 10.1126/sciadv.aaz5006

This PDF file includes:

Table S1
Figs. S1 to S10
References

Table S1. Changes in ITCZ mean position. Latitudinal change in the ITCZ position in the summer (June to September) following the eruption for TrNH and TrSH ensembles relative to the no-volcano case and for El Niño vs. La Niña conditions. The ITCZ position in the volcano and no-volcano simulations and the El Niño and La Niña cases is shown in brackets.

JJAS	$TrNH_{ENS01}$	$TrNH_{ENS02}$	$TrSH_{ENS01}$	$TrSH_{ENS02}$	NINO-NINA
$\Delta ITCZ_{GLOB}$	– 1.0°N<br>(6.6° – 7.6°)	– 0.9°N<br>(5.7 – 6.6°)	+0.9°N<br>(8.5° – 7.6°)	+0.3°N<br>(6.9° – 6.6°)	– 1.4°N<br>(6.6° – 8.0°)
$\Delta ITCZ_{PAC}$	– 0.5°N<br>(8.5 – 9.0°)	– 0.5°N<br>(6.1– 6.6°)	+ 0.7°N<br>(9.7 – 9.0°)	+0.5°N<br>(7.1– 6.6°)	– 2.4°N<br>(6.6° – 9.0°)

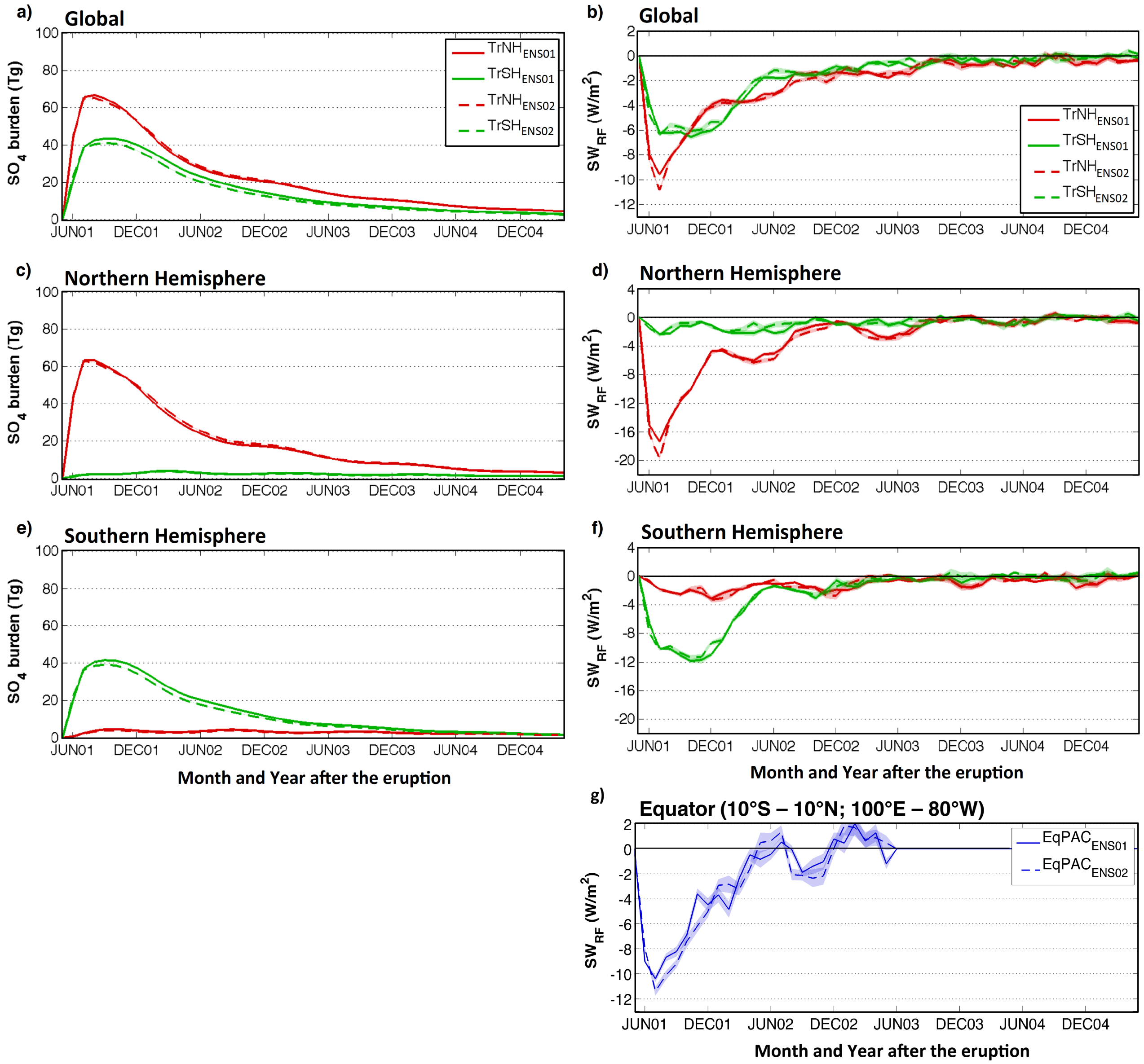

Figure S1. Sulfate aerosol burden and radiative forcing. Changes in Global (a, b), Northern Hemisphere (c, d) and Southern Hemisphere (e, f) sulfate aerosol burden (Tg) (left) and shortwave radiative forcing (W/m^2) at the surface (right) for TrNH and TrSH eruptions relative to no-volcano simulations. Changes in Equatorial Pacific radiative forcing for the EqPAC idealized experiments are shown in g. The shadings display the standard error of mean of each ensemble difference.

ENSO state relative to the climatology

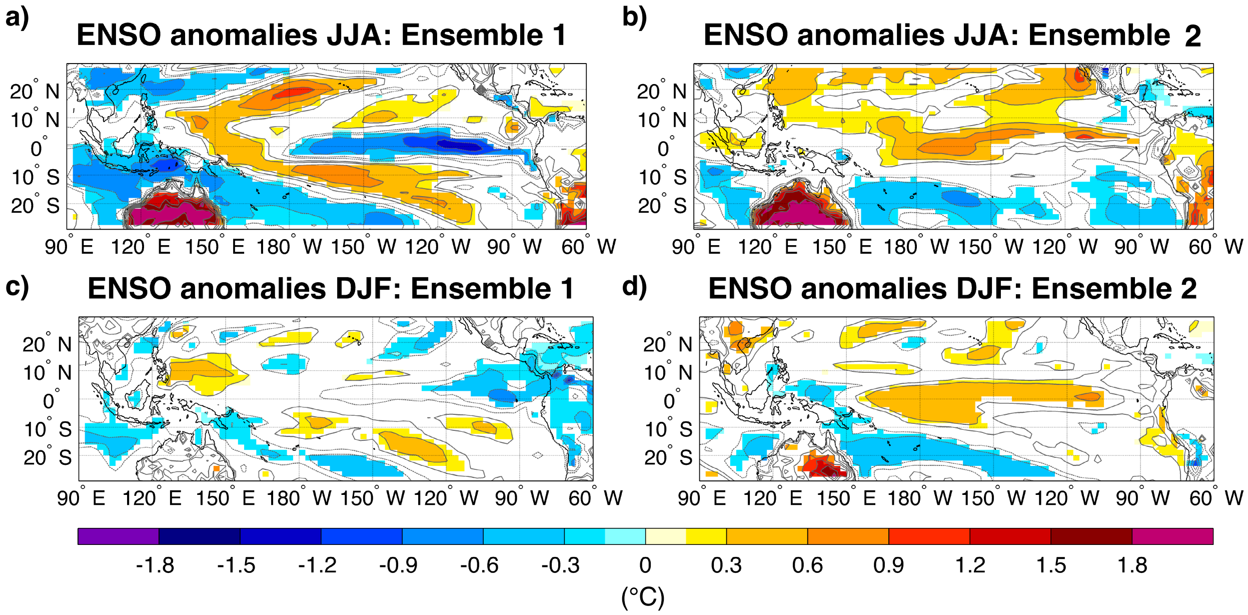

Figure S2: Changes in no-volcano ensemble surface temperature relative to the climatology. Changes in surface temperature (°C, shadings) in the first summer (June to August) (a, b) and winter (December to February) (c, d) following the start of the no-volcano ensembles relative to the reference historical simulation (1911-1964). Only anomalies that are significantly different at the 5% level using a local (grid-point) t test are shaded. The contours follow the colorbar intervals (solid for positive and dashed for negative anomalies; the zero line is omitted).

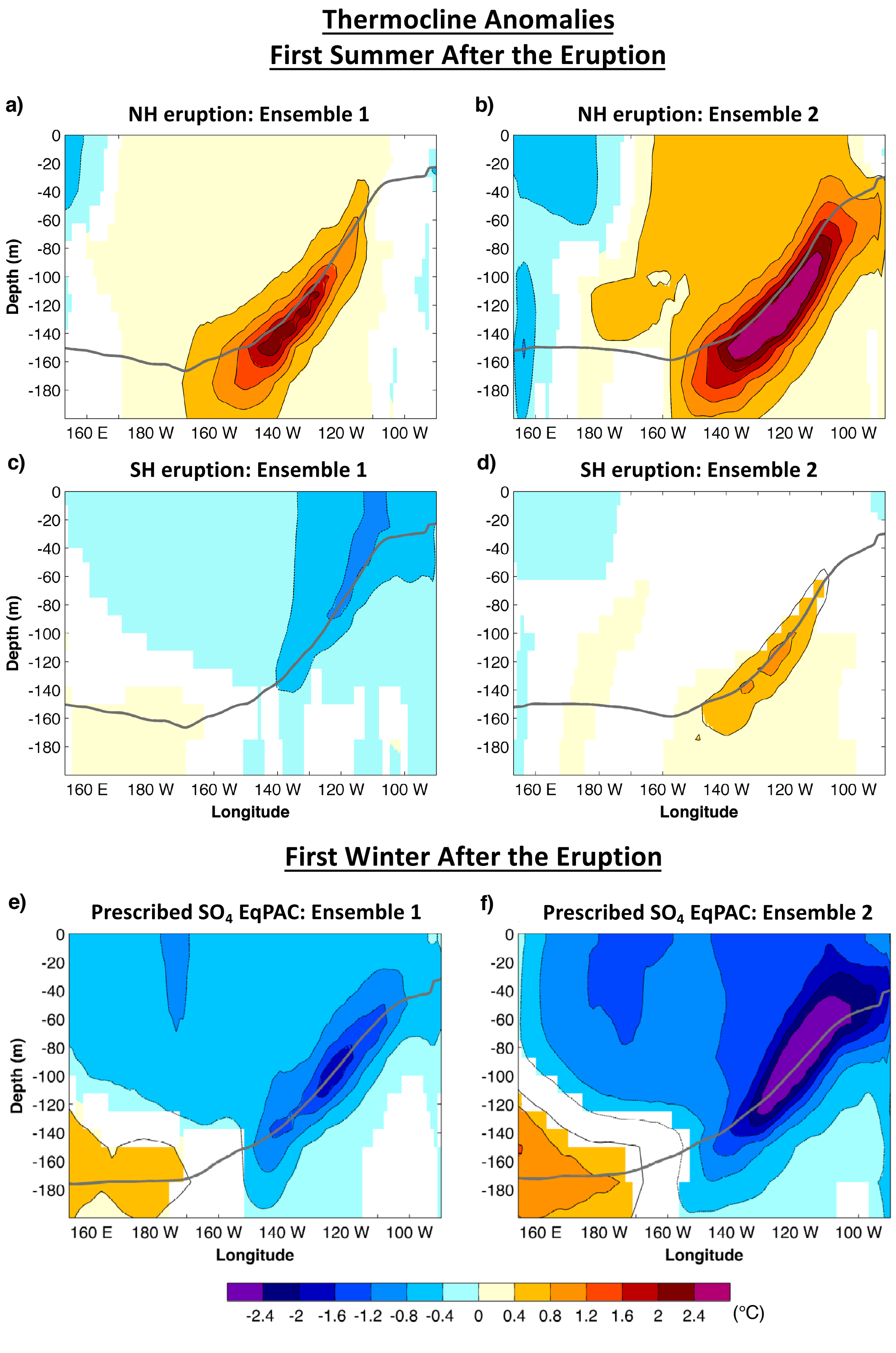

Figure S3. Thermocline anomalies. Ocean temperature (°C) anomalies in the Equatorial Pacific (5°S – 5°N) for the first summer (June to September) of the eruption for the TrNH (a, b) TrSH (c, d) experiments and the first winter (December to February) for the EqPAC simulations (e, f) relative to the no-volcano simulations. Only values that are significantly different at the 5% level using a *t* test are shaded. The contours follow the color bar intervals (solid for positive and dashed for negative anomalies; the zero line is omitted). The bold grey line shows the climatological thermocline depth for the no-volcano members (as defined using the 20°C isotherm).

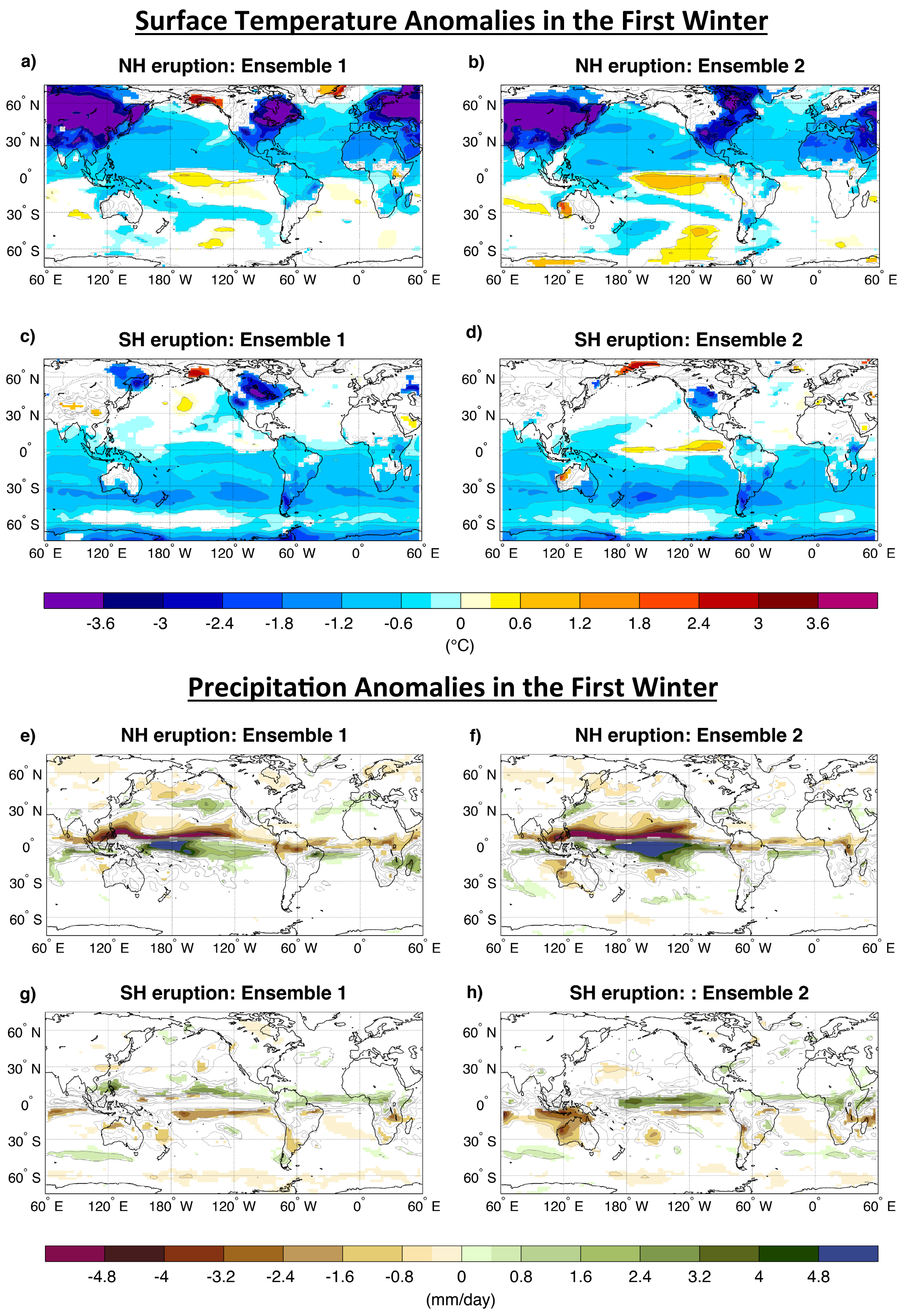

Figure S4. Changes in winter surface temperature and precipitation. Surface temperature (a-d) and precipitation (e-h) changes in the winter (December to February) following the TrNH (a, b, e, f) and TrSH (c, d, g, h) eruptions for each ensemble relative to the no-volcano simulations. Only values that are significantly different at the 5% level using a local (grid-point) *t* test are shaded. The contours follow the color bar intervals (solid for positive and dashed for negative anomalies; the zero line is omitted).

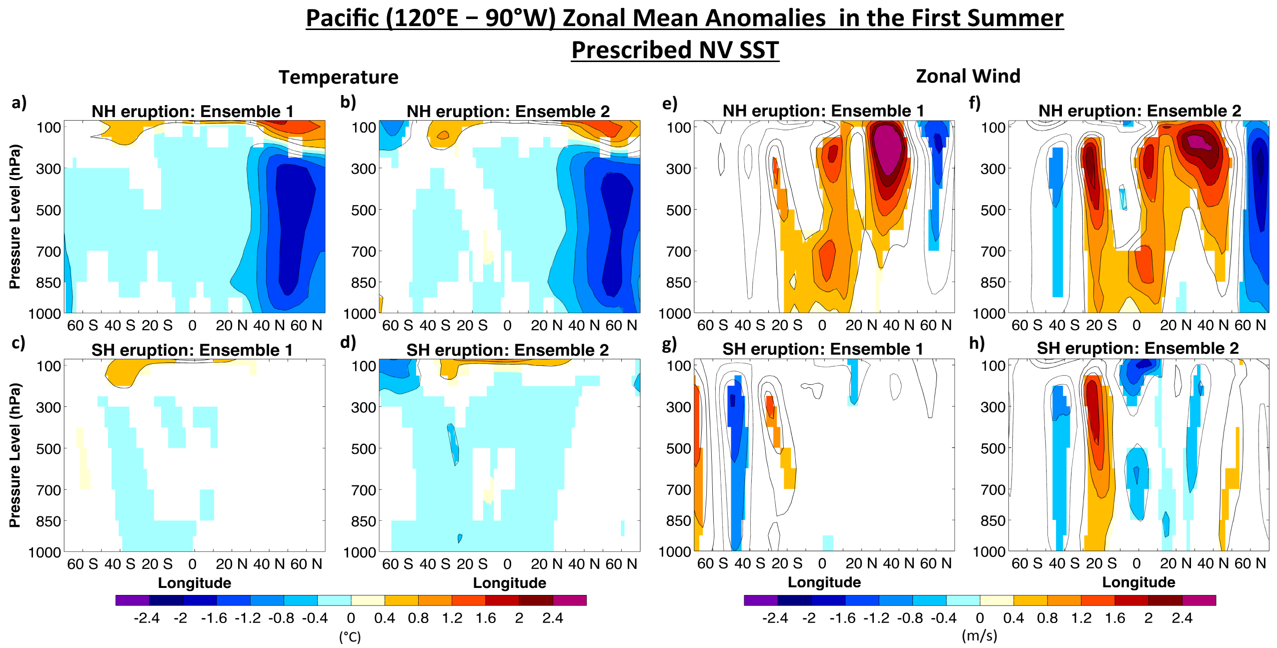

Figure S5. Pacific zonal-mean temperature and zonal wind anomalies for prescribed no-volcano SST experiments. Zonal-mean atmospheric temperature (a – d) and zonal wind (f – i) anomalies over the equatorial Pacific region (120°E – 90°W) in the summer (June to September) following the TrNH (a, b; f, g) and TrSH (c, d; h, i) eruptions for each ensemble using prescribed SST from the no-volcano experiments. Only values that are significantly different at the 5% level using a local (grid-point) *t* test are shaded. The contours follow the colorbar intervals (solid for positive and dashed for negative anomalies; the zero line is omitted).

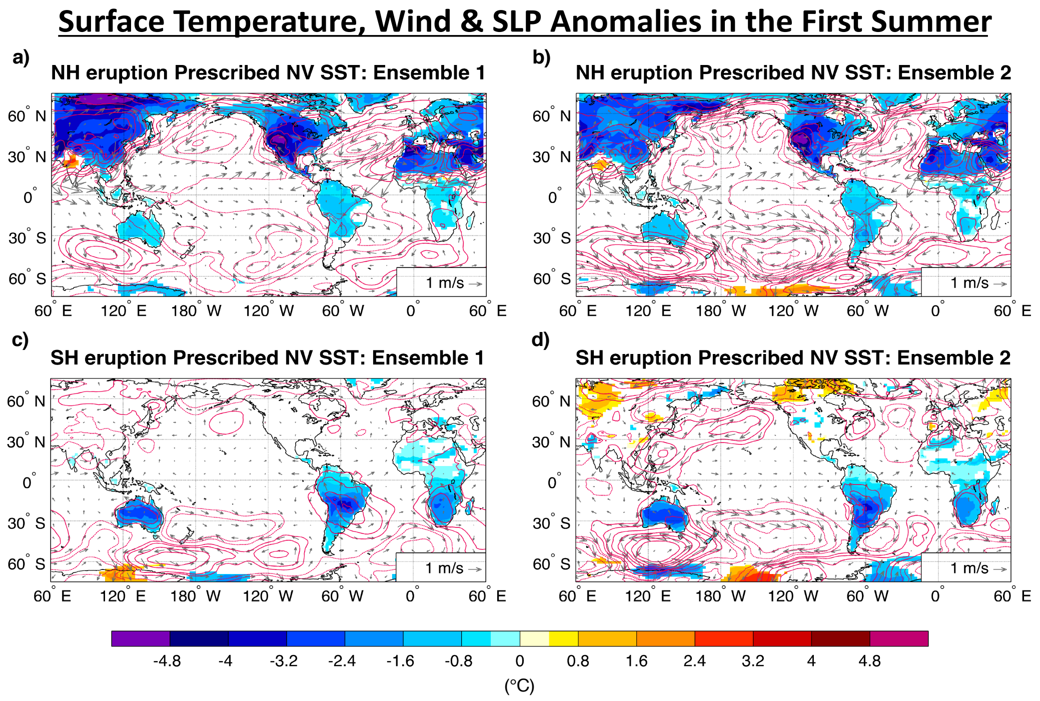

Figure S6. Changes in surface temperature, wind, sea level pressure for prescribed no-volcano SST experiments. Changes in surface temperature (°C, shadings), wind (m/s, arrows) and sea level pressure (hPa, contours) in the first summer (June to September) following the TrNH (a, b, e, f) and TrSH (c, d, g, h) eruptions for each ensemble using prescribed SST from the no-volcano experiments. Only temperature and precipitation values that are significantly different at the 5% level using a local (grid-point) *t* test are shaded. The contours follow the colorbar intervals (solid for positive and dashed for negative anomalies; the zero line is omitted).

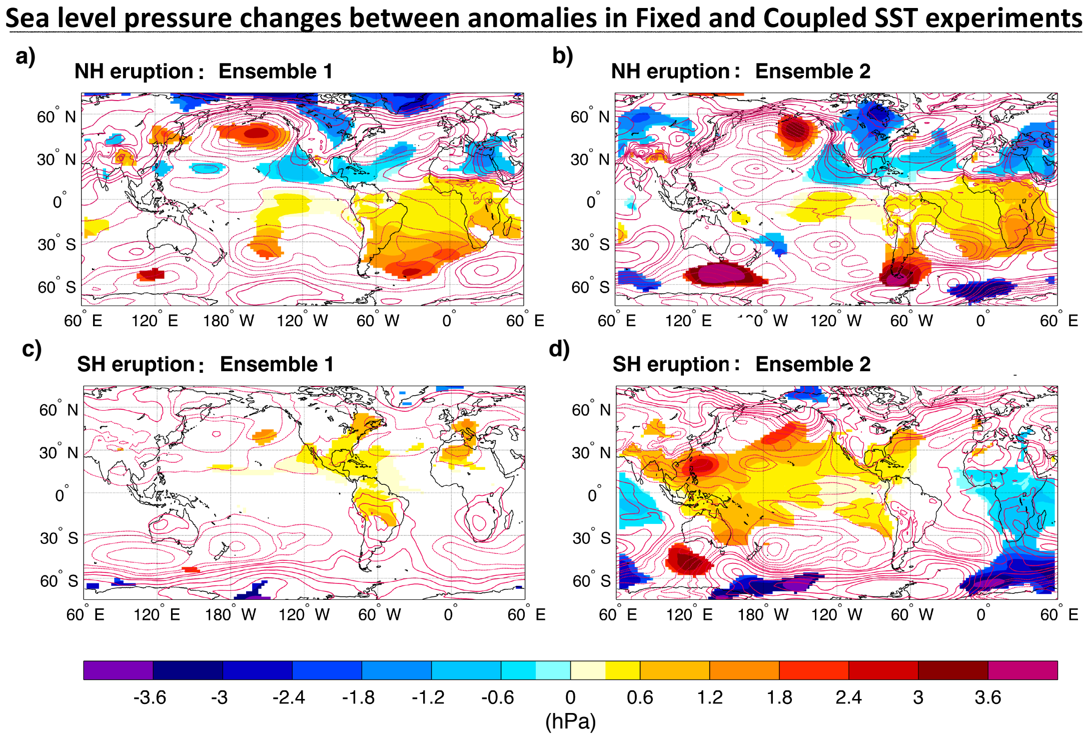

Figure S7: Changes in sea level pressure anomalies between prescribed and coupled SST experiments. Changes in sea level pressure (SLP) anomalies (hPa, shadings) between prescribed and coupled SST experiments in the first summer (June to September) following the TrNH (a, b) and TrSH (c, d) eruptions for each ensemble. Only anomalies that are significantly different at the 5% level using a local (grid-point) *t* test are shaded. The contours follow the colorbar intervals (solid for positive and dashed for negative anomalies; the zero line is omitted) and represent the coupled SLP anomalies.

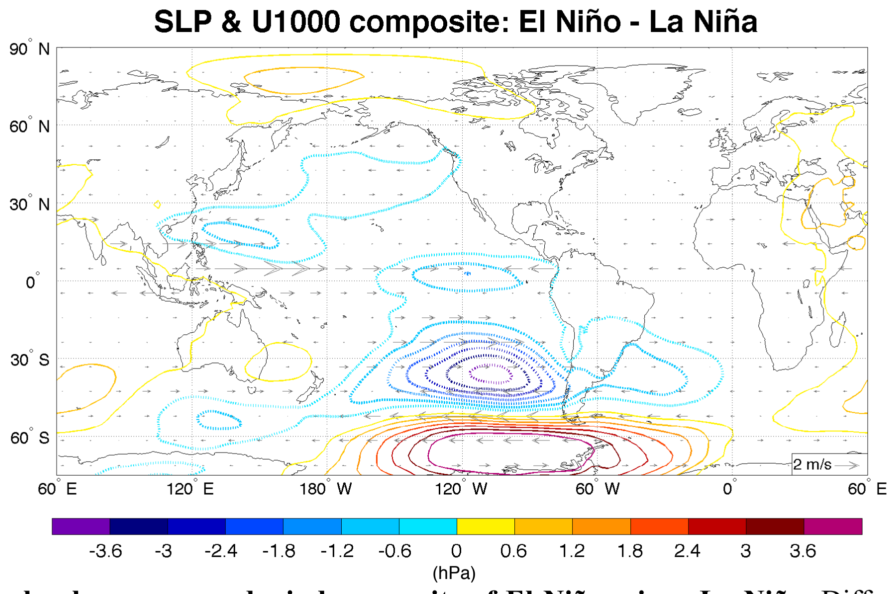

Figure S8. Sea level pressure and wind composite of El Niño minus La Niña. Difference between El Niño and La Niña composite of sea level pressure (a) and near-surface wind (b) for the summer (June to September – JJAS) preceeding the peak of ENSO events in the reference experiments. Only values that are significantly different at the 5% level using a local (grid-point) *t* test are shaded. The contours follow the color bar intervals (solid for positive and dashed for negative anomalies; the zero line is omitted).

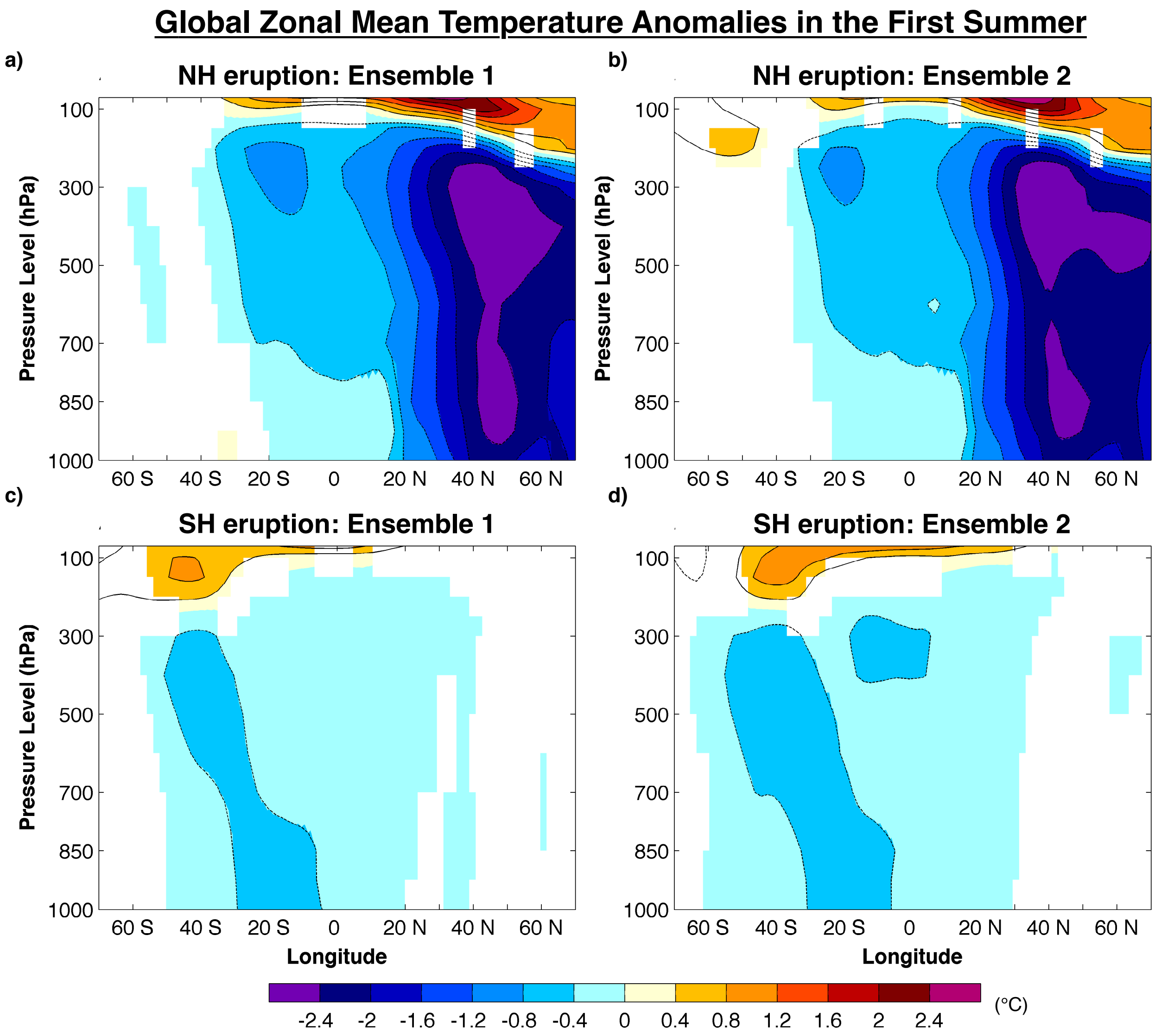

Figure S9. Global zonal mean temperature anomalies. Zonal mean atmospheric temperature anomalies over the entire globe in the summer (June to September – JJAS) following the TrNH (a, b) and TrSH (c, d) eruptions for each ensemble. Only values that are significantly different at the 5% level using a local (grid-point) *t* test are shaded. The contours follow the color bar intervals (solid for positive and dashed for negative anomalies; the zero line is omitted).

Model Validation

NorESM1-M does not include a module for the explicit treatment of stratospheric microphysics, therefore SO_2 / SO_4^{2-} interactions in the stratosphere goes through the tropospheric life-cycling scheme. Furthermore, NorESM1-M does not simulate growth by self-coagulation (coagulation of Aitken-mode particles combining to form larger particles). Self-coagulation is an important mechanism after an eruption when a massive amount of sulphate is injected, as described in *Pinto et al.* (*44*) and *English et al.* (*45*). Therefore, in NorESM1-M, SO_2 injection into the stratosphere leads to the formation of H_2SO_4 in a much finer mode compared to observed sizes as discussed in *Pausata et al.* (*43*). This can lead to significant biases in the climate response to volcanic eruptions due to the increased residence time of the aerosol particles as well as radiative forcing, especially when simulating strong tropical events where the injection height can reach the middle stratosphere up to 40 km asl. To overcome this shortcoming we lowered the injection height from 24 km to 18 km in our simulations and we validate our model against the Pinatubo eruption. When lowering the injection height, the Pinatubo simulation shows a better agreement with observations in terms of volcanic aerosol residence time and global

cooling (Fig. S10).

Specifically, to test the skill of NorESM1-M in simulating large tropical eruptions we run 5 simulations in which we injected 20 Tg of SO_2 at a mean altitude of 18 km mimiking the Pinatubo eruption. We aimed at reproducing a similar SO_4 peak concentration and e-folding time compared to observations. Injecting the SO_2 mostly between 15 and 21 km, the model show a SO_4 peak of ~22 Tg and an e-folding time of ~17 months (Fig. S8). Observational evidence indicates a sulfate aerosol production between 21 and 40 Mt (*46*) and an e-folding time between 12-14 months (*47–48*). Our model thus sits in the low-side of the observations for the SO_4 peak and slightly underestimates the SO_4 removal. The simulated global cooling for the Pinatubo eruption is around 0.4 °C, in agreement with observational and model-based estimates of 0.4-0.5°C (*49*). Therefore, we have adopted the same injection height for the Tambora experiments as for the Pinatubo eruption.

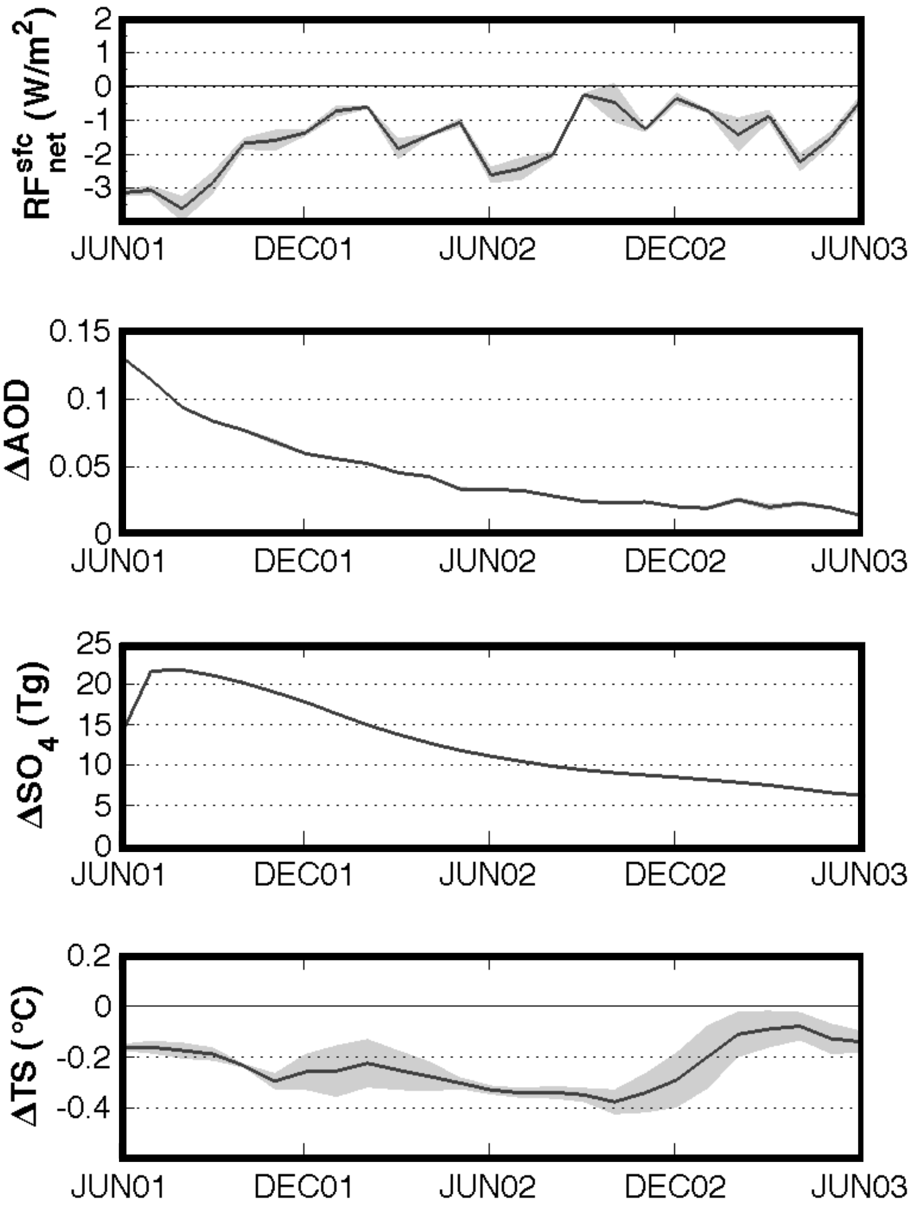

Figure S10. Global-average anomalies for Pinatubo eruption. Global-average anomalies of radiative forcing at the surface, aerosol optical depth, sulfate burden and surface temperature following a Pinatubo-like eruption simulated by NorESM1-M. Shading shows the approximate 95% confidence intervals (twice the Standard Error of the Mean) of the change seen in all 5 pairs of experiments performed.

REFERENCE AND NOTES

1. S. E. Zebiak, M. A. Cane, A model El Niño–Southern Oscillation. *Mon. Weather Rev.* **115**, 2262–2278 (1987).

2. C. F. Ropelewski, M. S. Halpert, Global and regional scale precipitation patterns associated with the El Niño/Southern Oscillation. *Mon. Weather Rev.* **115**, 1606–1626 (1987).

3. M. Sigl, M. Winstrup, J. R. McConnell, K. C. Welten, G. Plunkett, F. Ludlow, U. Büntgen, M. Caffee, N. Chellman, D. Dahl-Jensen, H. Fischer, S. Kipfstuhl, C. Kostick, O. J. Maselli, F. Mekhaldi, R. Mulvaney, R. Muscheler, D. R. Pasteris, J. R. Pilcher, M. Salzer, S. Schüpbach, J. P. Steffensen, B. M. Vinther, T. E. Woodruff, Timing and climate forcing of volcanic eruptions for the past 2,500 years. *Nature* **523**, 543–549 (2015).

4. K.-K. Tung, J. Zhou, The Pacific's Response to Surface Heating in 130 Yr of SST: La Niña–like or El Niño–like? *J. Atmos. Sci.* **67**, 2649–2657 (2010).

5. M. Khodri, T. Izumo, J. Vialard, S. Janicot, C. Cassou, M. Lengaigne, J. Mignot, G. Gastineau, E. Guilyardi, N. Lebas, A. Robock, M. J. McPhaden, Tropical explosive volcanic eruptions can trigger El Niño by cooling tropical Africa. *Nat. Commun.* **8**, 778 (2017).

6. S. McGregor, A. Timmermann, O. Timm, A unified proxy for ENSO and PDO variability since 1650. *Clim. Past.* **6**, 1–17 (2010).

7. J. B. Adams, M. E. Mann, C. M. Ammann, Proxy evidence for an El Niño-like response to volcanic forcing. *Nature* **426**, 274–278 (2003).

8 J. Li, S.-P. Xie, E. R. Cook, M. S. Morales, D. A. Christie, N. C. Johnson, F. Chen, R. D'Arrigo, A. M. Fowler, X. Gou, K. Fang, El Niño modulations over the past seven centuries. *Nat. Clim. Chang.* **3**, 822–826 (2013).

9. M. E. Mann, M. A. Cane, S. E. Zebiak, A. Clement, Volcanic and solar forcing of the tropical pacific over the past 1000 years. *J. Climate* **18**, 447–456 (2005).

10. M. Ohba, H. Shiogama, T. Yokohata, M. Watanabe, Impact of strong tropical volcanic eruptions on ENSO simulated in a coupled GCM. *J. Climate* **26**, 5169–5182 (2013).

11. N. Maher, S. McGregor, M. H. England, A. Sen Gupta, Effects of volcanism on tropical variability. *Geophys. Res. Lett.* **42**, 6024–6033 (2015).

12. S. Stevenson, B. Otto-Bliesner, J. Fasullo, E. Brady, "El Niño Like" hydroclimate responses to last millennium volcanic eruptions. *J. Climate* **29**, 2907–2921 (2016).

13. M. Zuo, W. Man, T. Zhou, Z. Guo, Different impacts of northern, tropical, and southern volcanic eruptions on the tropical pacific SST in the last millennium. *J. Climate* **31**, 6729–6744 (2018).

14. F. Liu, J. Li, B. Wang, J. Liu, T. Li, G. Huang, Z. Wang, Divergent El Niño responses to volcanic eruptions at different latitudes over the past millennium. *Clim. Dyn.* **50**, 3799–3812 (2018).

15. E. Predybaylo, G. L. Stenchikov, A. T. Wittenberg, F. Zeng, Impacts of a Pinatubo-size volcanic eruption on ENSO. *J. Geophys. Res. Atmos.* **122**, 925–947 (2017).

16. S. McGregor, A. Timmermann, The Effect of Explosive Tropical Volcanism on ENSO. *J. Climate* **24**, 2178–2191 (2011).

17. D. Zanchettin, C. Timmreck, H.-F. Graf, A. Rubino, S. Lorenz, K. Lohmann, K. Krüger, J. H. Jungclaus, Bi-decadal variability excited in the coupled ocean–atmosphere system by strong tropical volcanic eruptions. *Clim. Dyn.* **39**, 419–444 (2012).

18. S. Stevenson, J. T. Fasullo, B. L. Otto-Bliesner, R. A. Tomas, C. Gao, Role of eruption season in reconciling model and proxy responses to tropical volcanism. *Proc. Natl. Acad. Sci. U.S.A.* **114**, 1822–1826 (2017).

19. M. Hirono, On the trigger of El Niño Southern Oscillation by the forcing of early El Chichón volcanic aerosols. *J. Geophys. Res.* **93**, 5365–5384 (1988).

20. A. C. Clement, R. Seager, M. A. Cane, S. E. Zebiak, An ocean dynamical thermostat. *J. Clim.* **9**, 2190–2196 (1996).

21. J. Bjerknes, Atmospheric teleconnections from the equatorial Pacific. *Mon. Weather Rev.* **97**, 163–172 (1969).

22. F. S. R. Pausata, L. Chafik, R. Caballero, D. S. Battisti, Impacts of high-latitude volcanic eruptions on ENSO and AMOC. *Proc. Natl. Acad. Sci. U.S.A.* **112**, 13784–13788 (2015).

23. S. M. Kang, I. M. Held, D. M. W. Frierson, M. Zhao, The response of the ITCZ to extratropical thermal forcing: Idealized slab-ocean experiments with a GCM. *J. Climate* **21**, 3521–3532 (2008).

24. T. Schneider, T. Bischoff, G. H. Haug, Migrations and dynamics of the intertropical convergence zone. *Nature* **513**, 45–53 (2014).

25. C. M. Colose, A. N. LeGrande, M. Vuille, Hemispherically asymmetric volcanic forcing of tropical hydroclimate during the last millennium. *Earth Syst. Dynam.* **7**, 681–696 (2016).

26. M. Toohey, K. Krüger, U. Niemeier, C. Timmreck, The influence of eruption season on the global aerosol evolution and radiative impact of tropical volcanic eruptions. *Atmos. Chem. Phys.* **11**, 12351–12367 (2011).

27. M. Bentsen, I. Bethke, J. B. Debernard, T. Iversen, A. Kirkevåg, Ø. Seland, H. Drange, C. Roelandt, I. A. Seierstad, C. Hoose, J. E. Kristjánsson, The Norwegian Earth System Model, NorESM1-M – Part 1: Description and basic evaluation of the physical climate. *Geosci. Model Dev.* **6**, 687–720 (2013).

28. T. Iversen, M. Bentsen, I. Bethke, J. B. Debernard, A. Kirkevåg, Ø. Seland, H. Drange, J. E. Kristjansson, I. Medhaug, M. Sand, I. A. Seierstad, The Norwegian Earth System Model, NorESM1-M – Part 2: Climate response and scenario projections. *Geosci. Model Dev.* **6**, 389–415 (2013).

29. F. S. R. Pausata, C. Karamperidou, R. Caballero, D. S. Battisti, ENSO response to high-latitude volcanic eruptions in the Northern Hemisphere: The role of the initial conditions. *Geophys. Res. Lett.* **43**, 8694–8702 (2016).

30. T. A. Shaw, M. Baldwin, E. A. Barnes, R. Caballero, C. I. Garfinkel, Y.-T. Hwang, C. Li, P. A. O'Gorman, G. Rivière, I. R. Simpson, A. Voigt, Storm track processes and the opposing influences of climate change. *Nat. Geosci.* **9**, 656–664 (2016).

31. A. H. Butler, D. W. J. Thompson, R. Heikes, The Steady-State Atmospheric Circulation Response to Climate Change–like Thermal Forcings in a Simple General Circulation Model. *J. Climate* **23**, 3474–3496 (2010).

32. T. A. Shaw, A. Voigt, Tug of war on summertime circulation between radiative forcing and sea surface warming. *Nat. Geosci.* **8**, 560–566 (2015).

33. D. J. Vimont, J. M. Wallace, D. S. Battisti, The Seasonal Footprinting Mechanism in the Pacific: Implications for ENSO. *J. Climate* **16**, 2668–2675 (2003).

34. J. C. H. Chiang, D. J. Vimont, Analogous pacific and atlantic meridional modes of tropical atmosphere–ocean variability. *J. Climate* **17**, 4143–4158 (2004).

35. D. Zanchettin, C. Timmreck, M. Toohey, J. H. Jungclaus, M. Bittner, S. J. Lorenz, A. Rubino, Clarifying the relative role of forcing uncertainties and initial-condition unknowns in spreading the climate response to volcanic eruptions. *Geophys. Res. Lett.* **46**, 1602–1611 (2019).

36. R. B. Neale, J. Richter, S. Park, P. H. Lauritzen, S. J. Vavrus, P. J. Rasch, M. Zhang, The mean climate of the community atmosphere model (CAM4) in forced SST and fully coupled experiments. *J. Climate* **26**, 5150–5168 (2013).

37. A. Kirkevåg, T. Iversen, Ø. Seland, C. Hoose, J. E. Kristjánsson, H. Struthers, A. M. L. Ekman, S. Ghan, J. Griesfeller, E. D. Nilsson, M. Schulz, Aerosol-climate interactions in the Norwegian Earth System Model - NorESM1-M. *Geosci. Model Dev.* **6**, 207–244 (2013).

38. H. Sigurdsson, S. Carey, Plinian and co-ignimbrite tephra fall from the. *Bull. Volcanol.* **51**, 243–270 (1989).

39. M. Toohey, M. Sigl, Volcanic stratospheric sulfur injections and aerosol optical depth from 500 BCE to 1900 CE. *Earth Syst. Sci. Data.* **9**, 809–831 (2017).

40. C. C. Raible, S. Brönnimann, R. Auchmann, P. Brohan, T. L. Frölicher, H.-F. Graf, P. Jones, J. Luterbacher, S. Muthers, R. Neukom, A. Robock, S. Self, A. Sudrajat, C. Timmreck, M. Wegmann, Tambora 1815 as a test case for high impact volcanic eruptions: Earth system effects. *Wiley Interdiscip. Rev. Clim. Chang.* **7**, 569–589 (2016).

41. D. Zanchettin, M. Khodri, C. Timmreck, M. Toohey, A. Schmidt, E. P. Gerber, G. Hegerl, A. Robock, F. S. R. Pausata, W. T. Ball, S. E. Bauer, S. Bekki, S. S. Dhomse, Allegra N. Le Grande, G. W. Mann, L. Marshall, M. Mills, M. Marchand, U. Niemeier, V. Poulain, E. Rozanov, A. Rubino, A. Stenke, K. Tsigaridis, F. Tummon, The Model Intercomparison Project on the climatic response to Volcanic forcing (VolMIP): Experimental design and forcing input data for CMIP6. *Geosci. Model Dev.* **9**, (2016).

42. H. Bellenger, E. Guilyardi, J. Leloup, M. Lengaigne, J. Vialard, ENSO representation in climate models: From CMIP3 to CMIP5. *Clim. Dyn.* **42**, 1999–2018 (2014).

43. F. S. R. Pausata, A. Grini, R. Caballero, A. Hannachi, Ø. Seland, High-latitude volcanic eruptions in the Norwegian Earth System Model: The effect of different initial conditions and of the ensemble size. *Tellus, Ser. B Chem. Phys. Meteorol.* **67**, 26728 (2015).

44. J. P. Pinto, R. P. Turco, O. P. Toon, Self-limiting physical and chemical effects in volcanic eruption clouds. *J. Geophys. Res.* **94**, 11165 (1989).

45. J. M. English, O. B. Toon, M. J. Mills, Microphysical simulations of large volcanic eruptions: Pinatubo and Toba. *J. Geophys. Res. Atmos* **118**, 1880–1895 (2013).

46. P. B. Russell, J. M. Livingston, R. F. Pueschel, J. J. Bauman, J. B. Pollack, S. L. Brooks, P. Hamill, L. W. Thomason, L. L. Stowe, T. Deshler, E. G. Dutton, R. W. Bergstrom, Global to microscale evolution of the Pinatubo volcanic aerosol derived from diverse measurements and analyses. *J. Geophys. Res.* **101**, 18745 (1996).

47. A. Lambert, R. G. Grainger, J. J. Remedios, C. D. Rodgers, M. Corney, F. W. Taylor, Measurements of the evolution of the Mt. Pinatubo aerosol cloud by ISAMS. *Geophys. Res. Lett*. **20**, 1287–1290 (1993).

48. J. E. Barnes, D. J. Hofmann, Lidar measurements of stratospheric aerosol over Mauna Loa Observatory. *Geophys. Res. Lett.* **24**, 1923–1926 (1997).

49. I. Kirchner, G. L. Stenchikov, H.-F. Graf, A. Robock, J. C. Antuña, LClimate model simulation of winter warming and summer cooling following the 1991 Mount Pinatubo volcanic eruption. *Geophys. Res. Atmos.* **104**, 19039–19055 (1999).
